# Real Time Multipurpose Smart Waste Classification Model for Efficient Recycling in Smart Cities Using Multilayer Convolutional Neural Network and Perceptron

**DOI:** 10.3390/s21144916

**Published:** 2021-07-19

**Authors:** Ali Usman Gondal, Muhammad Imran Sadiq, Tariq Ali, Muhammad Irfan, Ahmad Shaf, Muhammad Aamir, Muhammad Shoaib, Adam Glowacz, Ryszard Tadeusiewicz, Eliasz Kantoch

**Affiliations:** 1 Department of Computer Science, Sahiwal Campus, COMSATS University Islamabad, Sahiwal 57000, Pakistan; ali.usman32332@gmail.com (A.U.G.); imransadiq0022@gmail.com (M.I.S.); ahmadshaf@cuisahiwal.edu.pk (A.S.); muhammadaamir@cuisahiwal.edu.pk (M.A.); mshoaib@cuisahiwal.edu.pk (M.S.); 2 Electrical Engineering Department, College of Engineering, Najran University Saudi Arabia, Najran 61441, Saudi Arabia; miditta@nu.edu.sa; 3 Department of Automatic Control and Robotics, Faculty of Electrical Engineering, Automatics, Computer Science and Biomedical Engineering, AGH University of Science and Technology, al. A. Mickiewicza 30, 30-059 Kraków, Poland; adglow@agh.edu.pl; 4 Department of Biocybernetics and Biomedical Engineering, Faculty of Electrical Engineering, Automatics, Computer Science and Biomedical Engineering, AGH University of Science and Technology, al. A. Mickiewicza 30, 30-059 Kraków, Poland; rtad@agh.edu.pl (R.T.); kantoch@agh.edu.pl (E.K.)

**Keywords:** deep learning, multilayer convolutional neural network (ML-CNN), multilayer perceptron, waste classification

## Abstract

Urbanization is a big concern for both developed and developing countries in recent years. People shift themselves and their families to urban areas for the sake of better education and a modern lifestyle. Due to rapid urbanization, cities are facing huge challenges, one of which is waste management, as the volume of waste is directly proportional to the people living in the city. The municipalities and the city administrations use the traditional wastage classification techniques which are manual, very slow, inefficient and costly. Therefore, automatic waste classification and management is essential for the cities that are being urbanized for the better recycling of waste. Better recycling of waste gives the opportunity to reduce the amount of waste sent to landfills by reducing the need to collect new raw material. In this paper, the idea of a real-time smart waste classification model is presented that uses a hybrid approach to classify waste into various classes. Two machine learning models, a multilayer perceptron and multilayer convolutional neural network (ML-CNN), are implemented. The multilayer perceptron is used to provide binary classification, i.e., metal or non-metal waste, and the CNN identifies the class of non-metal waste. A camera is placed in front of the waste conveyor belt, which takes a picture of the waste and classifies it. Upon successful classification, an automatic hand hammer is used to push the waste into the assigned labeled bucket. Experiments were carried out in a real-time environment with image segmentation. The training, testing, and validation accuracy of the purposed model was 0.99% under different training batches with different input features.

## 1. Introduction

According to a research, the amount global annual waste is considered to reach up to 2.2 billion tons in the next three to four years, which could cost round about USD 375 billion for waste management [1]. Another study of the Food and Agriculture Organization (FAO) of the United Nations shows that nearly 33% of the total food that is produced around the world for our daily consumption that is nearly 1.3 billion tons in weight, is lost or wasted [1,2]. Waste management is probably one of the major challenges that the world in the 21st century is about to face. A huge amount of the waste that could be recycled and could be used as raw material in many supply chains is dumped in landfills, which is very inefficient in countries’ economical perspective. This improper waste management could cause a huge adverse impact on the economy, environment and public health [3]. The waste recycling process is very significant both in terms of the economy and environment. According to the Environmental Protection Agency (EPA), municipal waste recycling is the second safest and most sound strategy for urban solid waste management [4]. In most developing countries, the local municipal corporations manually recycle the waste, which is very inefficient, time consuming and costly because the corporations have to hire a greater number of labor workers to work manually [5]. In developed countries, many techniques are used for efficient and less time-consuming waste management, including mechanical sorting or automatic sorting of waste according to its size and shape [6]. However, in developed countries, there is still a huge gap to improve the waste management at the local municipality level. The average amount of waste recycled in the European Union is around about 34% to 40%, which is not a good enough amount of recycling and much less than the target level, which is 75% in total [7]. The recent progress in the fields of image processing, deep learning and computer vision technology has changed the thinking about various aspects of daily life [8]. The idea of smart waste classification using images of waste and trash has a huge potential. Deep learning has provided a solid base for image recognition with a very consistent accuracy. The very popular image classifier, the convolutional neural network (CNN), is inspired by biological neural networks which consist of multiple layers and neurons in each layer are directly connected with the neurons in the next layer [9]. The advantage of using a convolutional neural network is that it provides independence from previous knowledge and less effort in feature extraction and design [9]. The convolutional neural network (CNN) has enjoyed a huge success in image recognition and classification [8]. The popularity and accuracy of the CNN for image classification has increased due to the huge availability of public data of images, high-speed GPUs and large-scale integrated systems for the processing of images and learning [10]. The hybrid approach of using a multilayer perceptron with the convolutional neural network is another great technique [11]. The sensor technology merged in the system is able to detect metal waste objects which are then classified into more specific classes [8]. Thus, image processing with deep learning has helped municipalities to get rid of manual waste classification. The smart image-based classification system has also provided solutions for mechanical and automatic size-based sorting of waste materials. The applications of smart waste classification are being utilized and giving good results [12].

In the adopted and implemented methodology, there is no human workforce required. Trash items can be directly thrown into the main container. A robotic arm picks and places trash items one by one onto the conveyor belt, a camera above the conveyor belt captures images of incoming trash items, and the camera stream is directly accessible via a Raspberry Pi (R-Pi). Pre-trained machine learning models are used to identify the type of trash item. After identifying the type of trash item, the item can be directly thrown into the respective trash bin. The trash management on the first level can be done without any human workforce. The system model at layer-0 does not store any data of collected garbage. The system status and other required data transmitted to layer-1 (the system monitoring layer) are stored on a temporary basis and records are kept of the overall system status. According to the available system resources, such as fast internet speed, all temporarily stored data are transferred to layer-2 (the cloud storage and analytical layer). Storing data in the cloud (layer-1) from multiple system end nodes is not possible. Internet speed and other system resources are needed at layer-0 of the system. Therefore, the local system monitoring layer (layer-1) is responsible for acquiring all the data from all the nodes. After collecting all the data, the data are then transferred to layer-1. The data stored in layer-1 are not permanent because after storing the data in the cloud, they are removed from layer-1. The transmission of data has no effect on the basic working of the system. Only these data will be used for analytical purposes, so some improvements in the system can be made without effecting the basic working of the system.

The paper is organized as follows:

In Section 2, a literature review about waste classification using machine learning is presented. Section 3 describes the methodology of our proposed system model. In Section 4, the high-level system model is described with all the layers and their responsibilities. In Section 5, all the results and comparisons are described with textual and graphical data.

## 2. Literature Review

The simplification of the waste classification process is necessary as technology has grown at a fast pace and most manual work has been reduced by adopting artificial intelligence techniques. A method is proposed for the classification of waste using the AI technique of deep learning [10]. The research has shown that, in the next 25 years, developing countries will be drastically affected by waste that is produced and not managed properly [13]. With the growing industrial capacity, the generation of solid waste, including paper, plastic, rubber, cotton and wood is also increasing. The only solution for waste management is dumping the waste in landfills. However, because of the landfills, the people residing around the landfills are badly affected with deadly diseases like cancer and these dumping practices are polluting the natural environment [14]. The proposed method is the combination of a support vector machine (SVM) and convolutional neural networks (CNNs) which deals with the separation and classification of the waste into various classes [10]. Due to the poor availability of trash datasets, a pre-trained model called ResNet-50, which is a type of convolutional network, is used in this study. Increasing the depth of the neural network gives better accuracy but generating a training error is more likely to occur, which leads to a problem called a vanishing gradient, which means the learning in earlier layers of the CNN is consequently dropped.

The Residual Network (ResNet-50) is a little different from a normal CNN and it can handle the problem of the vanishing gradient [15]. Different models have been developed previously for image classification problems [8,16,17]. The dataset used in this model is taken from [18]. The dataset consists of 1989 images which are divided into four classes: metal, plastic, paper and glass. The dimensions of all images are 512 × 384. The proposed methodology described in this paper is a combination of the ResNet-50 pre-trained model and a support vector machine [10]. The ResNet-50 pre-trained model was developed for image classification of 256 × 256 images with 1000 classes. The first layer of this ResNet-50 model is removed by setting the parameters “include-top is equal to false” and only few features come out of the model. These features are then given to the support vector machine and the SVM model predicts the class based on the features extracted using the ResNet-50 model. The dataset was divided into 8:2, meaning 80% for training and the remaining 20% for testing. The model was run for 12 epochs and it was noted that accuracy did not improve after 12 epochs. The model has an overall accuracy of 94.5%. The details of the methodology and results are given [10].

A framework for trash classification using transfer learning is proposed in [12]. The improvements in hardware components of the computer allow the deep learning models to provide solutions for various problems. Smart agriculture, business intelligence and energy consumption predictions are few examples of how machine learning and deep learning are facilitating and supporting improvements in daily life routines [19,20,21]. A deep neural network trash classification (DNN-TC) model is described in [12]. This DNN-TC framework is the advanced and improved version of the ResNext model proposed by [22], which is a deep learning model developed for image classification. DNN-TC uses ResNext as a base model and adds two more fully connected layers with 1024 output shapes. Data pre-processing is performed for images with high brightness, which is normalized by ranging the values of high brightness between 0 and 1. The horizontal flip technique and random crop technique are used to increase the number of input images in training and testing data batches. The training data are fed into the model architecture and the last output layer with the “log SoftMax” activation function predicts the class of the trash image. The DNN-TC model is firstly initialized by loading the ResNext model and then fine-tuned to make it compatible according to the image dataset and, lastly, the model is fitted and run for several epochs until the best accuracy is found. The experimental dataset used in the paper is from [18] and captured from a mobile device. The dataset has six different classes, including cardboard, plastic, metal, paper, glass and trash. Another dataset is also used in this paper, which consists of three classes: organic, inorganic and medical trash. The datasets and the results of experiments are described in [12].

Chu et al. proposed a multilayer hybrid deep learning system [11] for waste classification in urban areas. The multilayer hybrid model consists of several subsystems which combine to make a complete multilayer hybrid system. The core of the system is a convolutional neural network and a multilayer perceptron (MLP). The CNN is used to take images as input and trains on the whole dataset. Data pre-processing techniques are performed on the image before giving it to the model. The multilayer perceptron takes input data from sensors and inductors in the form of numerical computation. Pre-processing techniques are applied and then the data are given as input to the perceptron which predicts the class of the waste. The CNN is a very accurate model for image classification. The CNN consists of multiple layers, including convolutional layers, dense layers, pooling layers and output layers [23]. The multilayer perceptron is another model used for regression and classification problems. The layers between the input and output layer are called hidden layers. This model consists of one hidden layer with 10 neurons. The neurons of each layer take the input from the previous layers, sum all inputs and propagate them to the next layers as input. The proposed hardware in this model includes a high-resolution camera (model 0V9712), a bridge sensor and inductor. Multiple tests are performed to check the accuracy of the model. The complete details of the results are explained in [11].

## 3. Methodology

In this section, the methodology of smart waste classification system is described. The multilayer convolutional neural network works in a real-time environment to classify the waste type. Each layer of the convolutional neural network is described in a specific sequence so the input dimension matrix can be evaluated in the first phase. To work in a real-time context, all of the system modules work in a defined way so the output of a single module or multiple modules can be utilized so that the system can perform all the required tasks in a defined order. In our proposed system, the overall working structure and the cloud servers that are integrated with our system control the overall working of the system in case of failure.

### 3.1. Data Acquisition

To gather the input data, a camera is attached at the top of a conveyor belt. The conveyor belt moves trash to the buckets. Each bucket contains a specific category of waste. To separate the trash according to their category, we need to check each incoming item of waste. Therefore, to gather the appropriate information about the incoming waste item, we need some specific attributes of the data to identify the category. A high-definition camera is used to gather the input images of waste that is moving along the conveyor belt. The images are then directly fed to the machine learning model as the camera steam is bounded with a local control system.

### 3.2. Pre-Processing of Data

The pre-processing of the data can be carried out in a local control system. Incoming trash contains multiple types of waste, such as food waste, paper waste, medical waste, etc. The incoming stream contains the real-time images that contain some noise, e.g., blurry image portions, non-necessary objects, etc. The RGB image is converted to a grayscale model with alpha transparency so the background of the image becomes transparent. The major difference between simple grayscale and alpha grayscale is normalization of images with a transparent background. To reduce the blurriness, we used the sharpening kernels with a 2D filter. The image array is required to pass to the filter to apply the normalization. Before feeding the input image to the classifier, the image needs to be resized according to the classifier input layer.

### 3.3. Multilayer Neural Network

In our proposed machine learning model, we use a sequential model that is a stack of layers in which each layer has an exactly one tensor of input and exactly one tensor of output. A tensor is a multidimensional array of uniform type. Each layer is constructed with some set of attributes. A model summary is shown below. For the input image, first, some convolutional filters need to be applied. The 2D convolutional layers have some user-defined filters, and the filters ensure the number of number of kernels applied to the image. In our proposed model, we initially define 32 as the number of kernel filters. The kernel size in the input layer of the convolutional layer determines the dimensions of the kernel, and the kernel size must be an odd number tuple. This kernel tuple defines the size of the convolutional window. As such, padding in the convolutional window defines the padding that is applied to the input image, and the image is not at the very first layer. The same padding type is used. Activation function “linear” is used in the input convolution layer. As such, in the last layer (dense layer) activation function “SoftMax” is used. The activation function provides the convenience to the convolutional layer that is used after the convolution is applied. Python language is used for the creation of the overall system structure with the Py-Charm IDE. With the serial communication ports provided with the Raspberry Pi, data are transferred to transfer control commands.

### 3.4. System Model

In order to work in the real-time context, our proposed system model performs the sequential steps systematically according to the defined context with the use of a systematic approach of the sequential model for each system model activity.

### 3.5. Basic Structure

Each waste bucket contains only a specified type of waste; the main bucket contains the incoming trash. A robotic arm is used to grab a single trash item from the main bucket. Grabbing and placing the trash item on the conveyor belt is also carried out with a robotic arm. The conveyor belt rotates and passes by all of the buckets. Holders hold the incoming trash item if the category of the item matches with the category of the bucket. The main module of the system is trash item categorization. A pre-trained machine learning model takes the trash item image as input and categorizes the image. Between the input and categorization process, there is some pre-processing applied to the incoming data and after the final model result, some normalization techniques are applied to identify the data label. The output is a label that is transformed in the central control system to actuate the commands for the next flow of the system.

### 3.6. Working Structure

**Figure 1:**
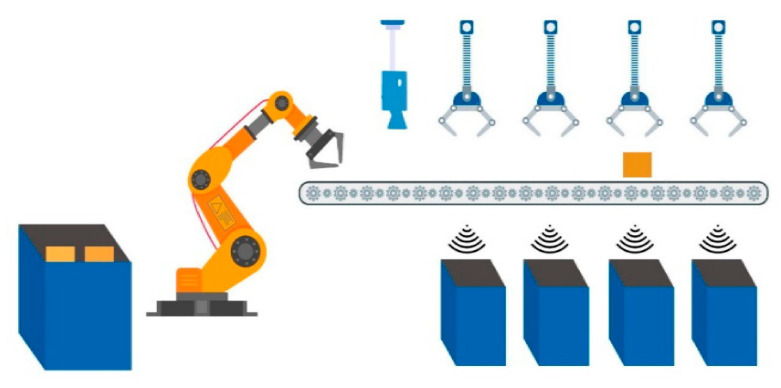
Graphical representation of picking waste items from the basket.

**Figure 2:**
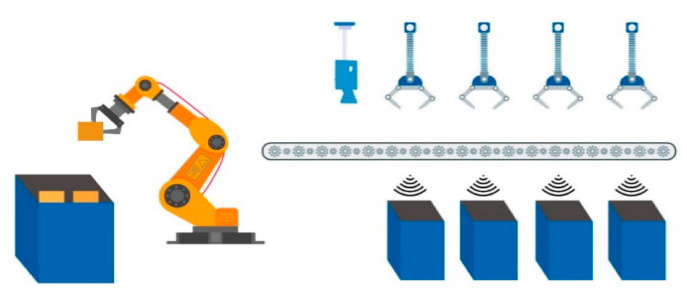
Taking a picture of waste item and classifying.

**Figure 3:**
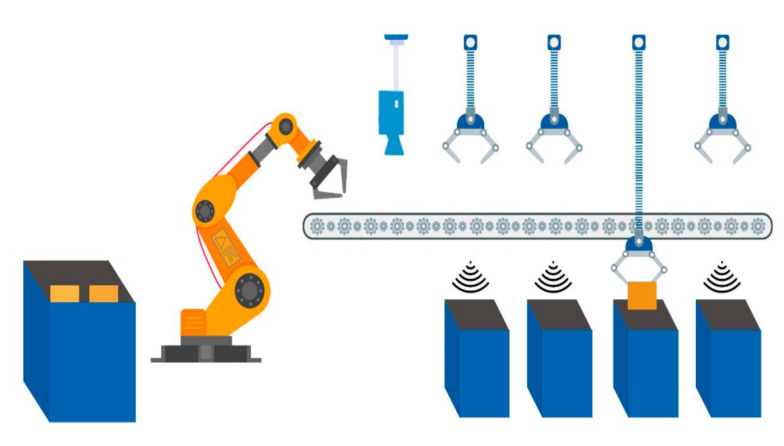
Transferring waste item from conveyor belt to bucket.

To define the main methodology of the overall system an overview of the system is provided. Incoming trash is directly thrown into the main trash bucket. Each trash item is placed on the conveyor belt one by one with the help of a robotic arm. A camera is placed at the top/start of the conveyor belt to acquire input images of the trash item. Basic sensors help to increase the efficiency of the system, for example, when the trash item passes directly below the camera, a simple distance sensor notifies the control system. Therefore, whenever a trash item passes below the camera, an image is captured and transformed in the control system. Image pre-processing and normalization of the image take place for the appropriate input for the pre-trained model. After categorization of the trash item, the actuation command is forwarded to the control system to throw the trash item in the matched trash bucket. The entire process is graphically represented in Figure 1, Figure 2 and Figure 3.

### 3.7. System Specification and Limitations

Design specifications and limitations of the implemented system model are described in Table 1.

## 4. Experimental Setup

**Figure 4:**
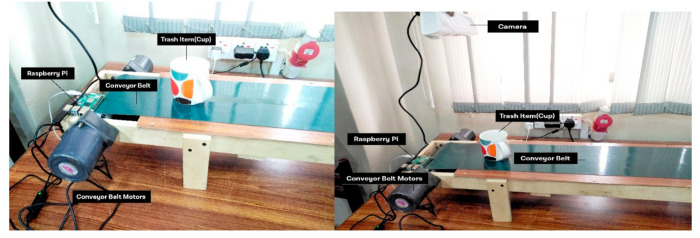
A real-time experimental view of classification cup.

**Figure 5:**
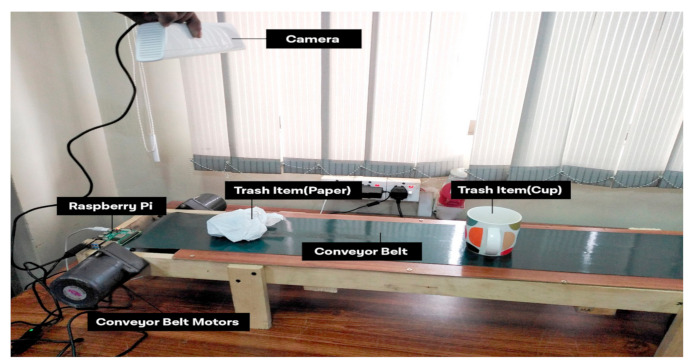
Experimental setup of waste classification.

The sustainable development of smart cities is purely based on the implementation of a consistent analytical model. The proposed system is built on the concept of central data processing and an analytical hub for smart control and processing of data related to multiple end nodes of the system. The data transmission among the system modules is based on reduced cost regarding load time and data delivery among them. There is no human workforce required at the system level. The sensory network and automated structure of the system is capable of actuating the task according to the requirement of the system. The experimental setup containing the conveyor belt and the GPU-based camera with a connective control structure is shown in Figure 4 and Figure 5.

### 4.1. Sensor-Level System Model

**Figure 6:**
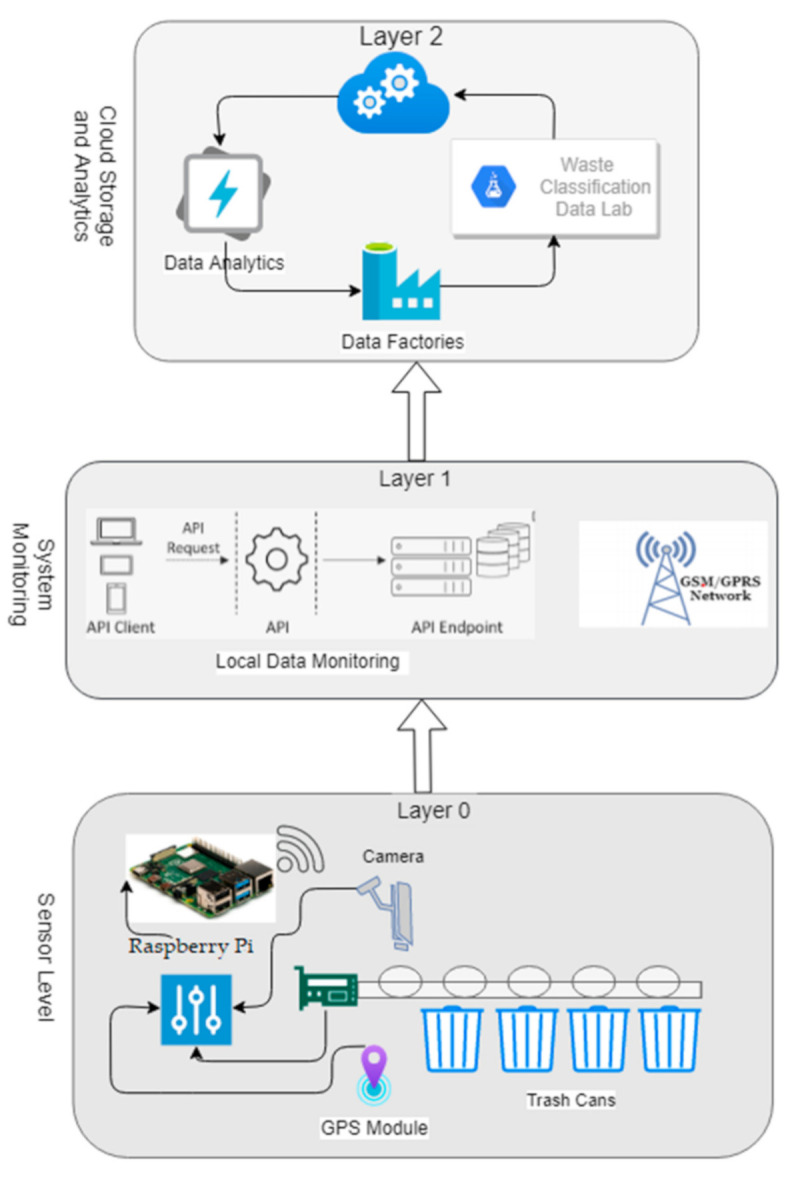
Experimental and system setup configurations.

**Figure 7:**
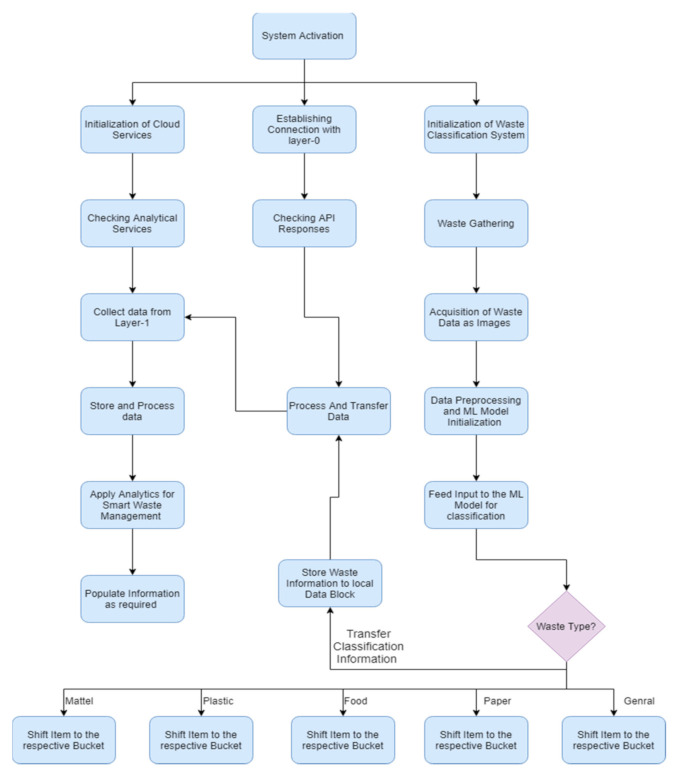
Experimental and system flow chart.

The distribution of the data and collection of garbage material can be handled at layer-0 of the system, as shown in Figure 6, and the system’s step by step execution process structure is given in Figure 7. The system deployment consists of the different sensor instruments (Wi-Fi, Raspberry Pi, microcontroller, GPS, camera, etc.). The responsibility of this layer is to collect and manage the garbage in an automated way without any human effort. Each bin contains trash items according to the system’s design (categorized items). The pre-trained machine learning model is used to recognize the category of the trash item, and with the help of conveyor belt the item then thrown in the assigned trash bin according to the category detected. The conveyor belt moves with the help of a microcontroller, on which it is totally dependent. For real-time tracking, the location and Global Positioning System (GPS) module are used and integrated with the Raspberry Pi. The location and status of the deployed system can be monitored anytime at all levels of the system. There is no data storage and analysis can be performed at this level. The required values and status of the system are transmitted to layer-1 after a specified time interval.

### 4.2. Local System Monitoring and Management

Accessing and monitoring the system status for the purpose of continuous and effective working of the system are necessary. Different APIs are created for different levels of the system. The responsibility of t layer-1 is to provide real-time tracking, mobility, interaction support services, coordination and synchronization. All these services are the responsibility of layer-1, as depicted in Figure 6, because transmission delay is a main factor for the cloud services when handling such a large amount of data from multiple nodes in real time. Data nodes installed at different locations of the city with a large distance between them cannot manage to transmit data in a real-time manner to the cloud (layer-2). The task of the sensor is to transmit data with a consistent speed and data format. Multiple data sensors or nodes with heterogeneous natures cannot transmit data directly to the cloud for processing in real time. This local monitoring system provides support to the end-level data nodes for sending and receiving required data. Using a web application interface, managing and streaming services of the system can be accessed with fast response and data mobility can be maintained. Integration with the sensor network is the responsibility of the local monitoring system. All these integration processes require good networking and communication support for effective and reliable communication.

### 4.3. Cloud Storage and Analytics

To store and manage a large amount of data and for analytical purposes, cloud computing is necessary. Processing power is the main reason for using the cloud as central storage, which can also help in working with analytics processes of the system. Due to different software services and the large data storage capability, data can be easily maintained and analyzed. Data from multiple sources with heterogeneous structures can be received. Maintaining data with heterogeneous structures and applying analytical approaches require high computational power. The communication between cloud storage and the local system monitoring system involves different communication requirements for smooth support for the end-level system structure. The detailed layer-2 structure is shown in Figure 6. The communication delay with this module of the system cannot affect all of the system. Layer-0 is totally independent of layer-2, because layer-0 needs no input from layer-2. Only the central or local monitoring system requires input from the cloud for updating the statistics for the system. Stored data in a heterogeneous structure require a homogeneous structure for further analytical processes. Data with a homogeneous structure used for the analytical process and different statistics are produced, which are useable in layer-1 of the system. The statistics describe the most used trash bins and most used locations. The garbage collecting service for selected nodes can be increased according to the need. All these types of information can be captured using analytical services of the system. Data factories maintain the analytical results with the actual state of the data for further use. The final results that are not necessary for further use are stored in the data lab that is the entire autonomous directory for the final analytical results.

## 5. Results and Discussion

**Figure 8:**
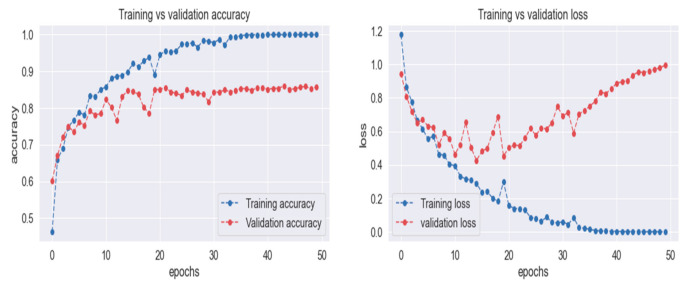
Training accuracy, validation accuracy, training loss and validation loss.

For the purpose of training and testing, real-time images are acquired for constructing the dataset. A total of 1241 images are used for training and 349 images used for testing purposes. The multilayer convolutional neural network is fitted for the training images in total of 100 epochs. Each epoch iterates with a total of 1241 images. Training loss is around 8.61 in the first epoch and it continuously decreases with each epoch and ends at around 0.1. Training accuracy ends at 0.99 that starts with 0.001 in the first epoch. The training accuracy and loss are shown in Figure 8, respectively.

During the testing phase, 35 image classes are generally mapped with five classes for better understating of the results. The mappings of all 35 classes are mapped as food, plastic, general, metal and paper. The model predicts the actual image types or trash type with their actual label, e.g., apple is a classification type in our dataset and finally mapped with food. Like food, the other mapping categories contain a total of 35 different categories. High precision, recall, F1-score and support are obtained at the time of testing, as shown in Table 2. Precision, recall, F1-score and support are described mathematically in Section 5.2.

To a total of 349 images, testing was applied and the highest accuracy of 0.99 was obtained. This is because of proper pre-processing and the use of precise image normalization techniques. The selection of model layers and filters at each layer was carried out after proper testing and input of the image. Table 3 contains the information of testing accuracy, macro average and weighted average with parameters of precision, recall, F1-score and support. Training loss and training accuracy show the model learning accuracy, as shown in Figure 8. Additionally, the testing accuracy shows how accurately the model classifies the incoming images corresponding to their labels.

### 5.1. Confusion Matrix

Misclassification of images by a trained machine learning model is defined as when an image of type A is wrongly classified as type B. The total misclassification and correct classification are shown in a confusion matrix in Table 4. A total of four images are wrongly classified, as shown. Three images of plastic are wrongly classified as metal (iron, copper, etc.) and paper. One image of paper is wrongly classified as food.

A graphical representation of a confusion matrix with the help of image plot libraries provides a better understanding of most the populated area of the graph that shows the most common class label. Above, in Table 4, we can see that the blue parts of the matrix mean no image is classified in that area.

### 5.2. Performance Evaluation Criteria

The performance comparison of the existing system model with our new enhanced system scheme and a machine learning model is described in the table below. The evaluation criteria for measuring the accuracy and other performance measures are given below in Equations (1)–(3).

(1) $$\mathrm{Precision}=\frac{TP}{\left(TP+FP\right)}\times 100\%$$

(2) $$\mathrm{Recall}=\frac{TP}{\left(TP+FN\right)}\times 100\%$$

(3) $$\mathrm{Accuracy}=\frac{\left(TP+TN\right)}{\left(TP+TN+FP+FN\right)}\times 100\%$$

(4) $$F1-\mathrm{Score}=\frac{2*\left(\mathrm{Recall}*\mathrm{Precision}\right)}{\left(\mathrm{Recall}+\mathrm{Precision}\right)}$$

Support = n (X) X is the target class
(5)

In the equations above, *TP*, *TN*, *FP* and *FN* stand for true positive, true negative, false positive and false negative, respectively. Let us take an example of two different waste objects from this study to understand the terms mentioned above. Our trained model is trained on two classes, metal and paper. After training completion, if we feed an image of metal to the model for prediction and the model correctly identifies the image as a metal object, that is called a true positive. However, if the model classifies the image of metal as paper, then it will be defined as a false positive. The same occurs in the case of a true negative, if the model correctly classifies an image of paper as paper, and a false negative occurs if the model incorrectly classifies the image of paper as an image of metal.

With the implementation of the proposed model, 99% accuracy is achieved both for the ML-CNN and MLP. As shown in the comparison provided in Table 5, this high accuracy was obtained by using a high-quality camera with GPU support and a high-fidelity image depth. High-depth images support finding the object skeletons and the generation of the depth map for the trash items. The training process of the machine learning model with high-quality images and testing with a high transfer rate and quality of the images provides the ability to achieve this accuracy. Machine learning models (ML-CNN and ML perceptron) with a refined sequence of layers and activation functions update the weights of network by keeping the previous layer output in mind that is used for the back propagation of the ML perceptron with a linear activation function for the classification process.

The main focus behind the implementation of this system is to classify garbage in a real-time context. It involves a GPU-based camera with a high depth of output images and a GPU-based central unit for acquiring and transferring control signals to the base system units and layer-3 of our proposed and implemented structure. The data are transferred from the base unit to the central unit and then to the cloud database for storing the required data and analytical processes. This scheme of the system results in a very low error rate obtained by using refined machine learning models and system modules with enough support to work in a specified manner.

## 6. Conclusions

The traditional waste classification techniques are manual, very slow, inefficient and costly. Therefore, automatic waste classification and management are essential for cities that are being urbanized for the better recycling of waste. In conclusion, a smart waste classification model is presented with all of its structures and the running environment that are essential to deploy the model in a real-world environment. The model is necessary with respect to advanced technologies, especially the concept of smart cities. The manual separation of waste and clustering of all waste items are unsuitable for smart cities. The smart waste classification model is the basis for the multilayer convolutional deep learning model with some physical requirements, including a system with a conveyer belt, a pushing hammer and garbage baskets which will collect the garbage items pushed by the hammer according to the class of the waste item. The dataset used in this model is generated in a real-time environment with a high-resolution camera. Each waste item moving on the conveyer belt is firstly captured through a camera placed at a fixed position and then the picture of the waste item is given as an input to the trained model. The model predicts the class of the waste item and when the item reaches the relevant basket, it is thrown into it by activating the correct hammer. The trained model, with the testing data, gives an accuracy of 0.99% which is highly reliable and consistent. The deployment of this model will surely not only be helpful in fast real-time clustering of waste items but also allow recycling companies to use these waste items for the production of new items. The effect of this model on climate change will be very positive as less waste will be sent to landfills which will cause less burning of waste and garbage.

## Figures and Tables

**Table 1:** Design specifications and limitations of implemented system model.

Part Name	Details	
Robotic Arm	Task	The core functionality of this robotic arm is to pick a single item from a trash bucket and place it on the conveyor belt.
	Specification	It can pick and place trash item of around 2 KG. The arm is capable of shifting 15 items per minute.
	Limitations	Claw of arm is capable of picking items of 12 cm to 20 cm. The weight limitation of arm is 2 KG.
Conveyor Belt	Task	Functionality of moving conveyor belt is to move within a specific speed range defined as 12 complete rotations in a minute.
	Specification	Trash items will be moved after classification to their corresponding buckets. It can carry items of around 2 KG in weight.
	Limitations	Speed of the movement cannot be increased in this step, because this architecture is also configured with the classification process, so the specified time interval is also added to the total time of rotation. Weight limitation cannot be increased in this step, because this will also slow down the belt movement process. Using heavy motors and refined mechanical model can increase the weight limitation.
Camera	Task	Capturing images of the trash items and transferring them to the connected Raspberry Pi module for classification process.
	Specification	It has GPU support that increases the overall image capturing and transferring process.
	Limitations	There is no limitation for this module of the system.
Raspberry Pi	Task	A tiny processor providing control structure for the classification process. With connected local server configured on Jetson Nano, it can transfer required data to the other module of the architecture for the next process.
	Specification	Can run machine learning model for classification process. A portable device that is easy to program and configure.
	Limitations	Low computation power can affect the performance of the local system, not over-line.

**Table 2:** Individual results with the basic performance measures.

	Precision	Recall	F1-Score	Support
Food	0.99	1.00	0.99	83
Plastic	1.00	0.95	0.98	62
General	1.00	1.00	1.00	72
Metal	0.99	1.00	0.99	92
Paper	0.95	0.97	0.96	40

**Table 3:** Cumulative results.

	Precision	Recall	F1-Score	Support
Accuracy			0.99	349
Macro avg.	0.99	0.99	0.99	349
Weighted avg.	0.99	0.99	0.99	349

**Table 4:** Confusion matrix.

	Food	Plastic	General	Metal	Paper
Food	83	0	0	0	0
Plastic	0	59	0	1	2
General	0	0	72	0	0
Metal	0	0	0	92	0
Paper	1	0	0	0	39

**Table 5:** Comparison of previous models in terms of accuracy.

Reference	Dataset	Model	Precision	Recall	Accuracy	No. of Groups of Classes
(Yinghao Chu et al., 2018)	Self-generated	CNN	88.6%	6.8%	87.7%	2
		MHS	97.1%	92.3%	91.6%	
Our Model	Self-generated	MLP	98.1%	8.4%	99%	5
		CNN	98%	98%	99%	

## Data Availability

There is no publicly available dataset used in this research paper.
